# OA10.03. Improved activity, participation, and quality of life for individuals with chronic stroke following an 8-week yoga intervention

**DOI:** 10.1186/1472-6882-12-S1-O39

**Published:** 2012-06-12

**Authors:** M Van Puymbroeck, A Schmid, K Miller, N Schalk

**Affiliations:** 1 Indiana University, Bloomington, USA; 2 Roudebush VAMC/ Indiana University, Indianapolis, USA; 3 Heartland Yoga, Indianapolis, USA

## Purpose

The purpose of this study was to determine if participation in an 8-week yoga program improved activity, participation, and quality of life in individuals with chronic stroke.

## Methods

For this pilot study, individuals were randomized 3:1 into the experimental group or wait list control. Forty-seven individuals were recruited into the study, with 37 individuals randomized to the yoga arm, and 10 randomized into the control group. Twenty-nine individuals completed the yoga intervention (22% attrition). The yoga intervention occurred twice per week for 1 hour each session, and was designed for individuals with chronic stroke (>6 months post event). All yoga classes were led by a registered yoga therapist. To measure activity and participation, the ICF Measure of Participation and Activity (IMPACT) subscales were utilized (lower scores indicate higher activity or participation). To measure quality of life, the Stroke Survivor Quality of Life (SSQOL) scale was utilized. Paired t-tests were utilized to compare the baseline and 8-week scores on each of the measures for both groups.

## Results

The mean age of the participants was 64, most were male (76%), married (47%), white (63%), and had some college education (42%). For individuals in the yoga group (n=29), activity improved (t=2.45, p=.02), participation improved (t=2.10, p=.045), and quality of life improved (t=-2.187, p=.04). For those in the control group (n=9), activity, participation, and quality of life did not statistically significantly improve over the 8-week period.

## Conclusion

The 8-week yoga intervention for individuals with chronic stroke resulted in improved activity, participation, and quality of life; while those in the control group did not see improvements in these areas. These findings support future research in these areas to determine the mechanisms from yoga that improved activity, participation, and quality of life for individuals with chronic stroke.

